# Hematological indexes and iron status in pregnant mares

**DOI:** 10.5194/aab-66-197-2023

**Published:** 2023-07-27

**Authors:** Katiuska Satué, Esterina Fazio, Deborah La Fauci, Pietro Medica

**Affiliations:** 1 Department of Animal Medicine and Surgery, Faculty of Veterinary Medicine, CEU Cardenal Herrera University, 46115 Valencia, Spain; 2 Department of Veterinary Sciences, Veterinary Physiology Unit, Polo Universitario Annunziata, Viale Palatucci 13, 98168 Messina, Italy

## Abstract

During pregnancy, iron requirements are increased to meet optimal placental and fetal growth and the expansion of the maternal red-cell mass and to prevent complications related to the mother's iron deficiency anemia. Red-cell parameters and iron status provide consistent additional information for diagnosis of iron deficiency conditions. The aim of this study was to evaluate the serum iron status and its relation to hematological indexes in pregnant mares. Blood samples were taken from 31 Spanish Purebred mares over 11 months of pregnancy. Concentrations of iron (Fe), ferritin (Ferr), transferrin (T), and total iron-binding capacity (TIBC) increased significantly and unsaturated iron-binding capacity (UIBC) decreased as the pregnancy progressed without changes in red blood cell (RBC) count, hemoglobin (HB) concentration, packed cell volume (PCV), and transferrin saturation (TSAT). Fe and Ferr were positively correlated ( r=0.21 ). Fe and T ( r=0.69 ) and Fe and TSAT ( r=0.94 ) were positively correlated, and Fe and UIBC were negatively correlated ( r=-0.69 ). T and TIBC were positively correlated ( r=1.00 ). Pregnancy in the Spanish Purebred mare is characterized by a progressive increase in Fe, Ferr, T, and TIBC and a decrease in UIBC without modification in hematological indexes. Hematological parameters and iron status seem to indicate a sufficiency for Fe transport and its related mobilization and utilization during gestation in Spanish Purebred mares.

## 1 Introduction

Physiologic demand for iron (Fe) increases during pregnancy to meet plasma volume expansion and fetal iron requirements and to promote increased red-cell mass and feto-placental unit growth (Fisher and Nemeth, 2017). The World Health Organization (WHO) emphasizes that Fe deficiency and anemia during pregnancy represent a major public health problem, resulting in adverse effects for both the mother and the newborn, including alterations of oxygen supply to the placenta and fetus, interference with intrauterine growth, pre-term labor, and increased risk of bleeding, sepsis, and maternal and perinatal mortality (WHO, 2021). It is estimated that one-fifth of women with uncomplicated pregnancies develop anemia in the third trimester and approximately half of women without anemia in the second trimester develop anemia during the third trimester. Indeed, in women of childbearing age, Fe deficiency is extremely common, since Fe loss due to menstruation and pregnancy cannot be fully compensated for by increased Fe intake (Camaschella, 2019). Therefore, early prediction of anemia risk in pregnant women is clinically important to allow for early intervention such as Fe supplementation (Noshiro et al., 2022).

Common profiles of iron status include red blood cell (RBC) count; hemoglobin (HB) concentration; packed cell volume (PCV); serum iron (Fe), ferritin (Ferr), and transferrin (T) concentrations; T saturation (TSAT); total iron-binding capacity (TIBC); and unsaturated iron-binding capacity (UIBC). Fe binds T for transport in the blood, TIBC reflects the amount of T available for Fe binding (Harvey, 2012), TSAT reflects the percentage of serum Fe relative to the TIBC of available T, and UIBC reflects the ability for unsaturated Fe fixation (Åsberg et al., 2012, 2013; AL-Hamdani, 2022).

Serum Fe and TIBC levels for the calculation of TSAT < 20 % suggest an inadequate supply of Fe for HB synthesis and RBC production (Auerbach and Adamson, 2016). Serum Fe, represented as T-bound Fe available for incorporation into HB in developing erythroblasts in the bone marrow (Auerbach and Adamson, 2016), ranges from 26 to 348 µ g dL ${}^{-1}$ , and Ferr equates to less than 90 % (Larsson et al., 2008), indicating that most patients in the second trimester suffer from Fe deficiency. In addition, HB existing in the first trimester is capable of efficiently distinguishing between women, with an elevated risk of anemia during the third trimester in women. Thus, HB levels < 12.6 g dL ${}^{-1}$ during the first trimester lead to the development of anemia in 34 % of women, while HB levels ≥ 12.6 g dL ${}^{-1}$ lead to the development of anemia in only 6.7 % of women during the third trimester of pregnancy (Noshiro et al., 2022). In addition, Young et al. (2012) reported an increase in TIBC and UIBC during pregnancy; this was more pronounced in UIBC as the pregnancy progressed to term. Several mechanisms, such as hemodilution, pre-conception dietary intake, vitamin–mineral deficiencies like hypocobalaminaemia and hypofolataemia, and concurrent inflammatory conditions, jointly contribute to the development of gestational anemia in women (Scholl, 2005; Mei et al., 2011). As in women, in some animal species such as sows (Bhattarai et al., 2019) and bitches (Nivy et al., 2019), pregnancy also leads to anemia. Indeed, compared to mid-gestation, serum Fe and TSAT increase at the end of gestation in pregnant bitches, although RBC decreases (Nivy et al., 2019).

Although several studies on Quarter Horse, Thoroughbred (Harvey et al., 1994; Faramarzi et al., 2018), Marchador Mangalarga (Silva et al., 2019), Standardbred (Mariella et al., 2014), and Andalusian mares (Faramarzi et al., 2018) have reported decreased erythrocyte parameters, in Carthusian pregnant mares (Satué et al., 2009) no changes have been revealed during gestation. Despite the existing information about the hematological variables, the parameters indicative of the iron status are very scarce and fragmentary, having detected increases in Fe and Ferr in pregnant (Montesinos and Satué, 2013) and in HB and Fe in peripartum mares (Fazio et al., 2019). What is more, the existence of estrogen–iron axes in both pregnant and cyclic mares was recently recorded (Satuè et al., 2023a, b) along with related changes in hepcidin, ferritin, and iron homeostasis (Satue et al., 2023c, d).

To the authors' knowledge, changes in iron status parameters, such as Fe, Ferr, TIBC, T, TSAT, and UIBC, have been reported under physiological conditions represented by exercise and pathological conditions like insulin resistance and hemochromatosis. Regarding exercise, an increase in Fe, Ferr, T, TIBC, and UIBC after training in Thoroughbreds (Inoue et al., 2005; Abramovitc et al., 2014; Assenza et al., 2016) and in Italian Saddlebred jumpers (Piccione et al., 2017) has been reported. During insulin resistance (Kellon and Gustafson, 2020), serum Fe and Ferr concentrations and hemochromatosis due to chronic Fe overload and TSAT are markedly increased (Theelen et al., 2019).

The objective of this study was to evaluate the relationship between the erythrocyte parameters (RBC, HB concentration, and PCV) and the markers of iron status (serum Fe, Ferr, T, TSAT, TIBC, and UIBC values) from the 1st to the 11th month of pregnancy in healthy Spanish Purebred mares.

## 2 Materials and methods

### 2.1 Mares

A total of 31 healthy reproductive Spanish Purebred mares, aged 4–17 years old, were studied during the 1st to the 11th months of pregnancy. The study was performed in one horse-breeding station near Valencia, Spain (39 ${}^{\circ }$ 29 ${}^{\prime }$ N, 0 ${}^{\circ }$ 42 ${}^{\prime }$ W; 200 m above sea level). The criteria for the inclusion of mares in this research were as follows: absence of antibiotic or glucocorticoid treatments within the sampling period, absence of vitamins or iron supplements for 3 months prior to the examination, and submission to an appropriate vaccination and deworming program. All mares were bred under the same handling conditions, receiving the same reproductive management. During the sampling period, the mares' diet was a combination of fiber and compounded food, divided into twice-daily administrations. The fiber consisted of 2–3 kg of alfalfa hay and straw, with natural iron contents of 30 to 1200 mg kg ${}^{-1}$ and 30 to 300 mg kg ${}^{-1}$ , respectively. Mares based their diet on hay ad libitum ( ∼ 7.5 kg) and 4 kg of concentrated feed based on barley, oats, corn, and wheat during the first 8 months of gestation. From this moment until the moment of delivery, they were supplemented using a special feed (Pavo®) at a dose of 0.42 kg per 100 kg of live weight per day. Water was ad libitum.

### 2.2 Blood samples

Venous blood samples were obtained from 31 reproductive Spanish Purebred mares once a month during the 11 months of gestation. All the samples were taken before the meal administration. All mares became pregnant in late February, March, and early April. The mean pregnancy length was paired to 330.1±10.1 d. The last blood samples were taken 7 to 15 d before parturition. After physiologically foaling, all mares were in the lactation phase and had foals on their sides.

Blood samples were always collected by jugular venipuncture between 08:00 and 11:00 UTC +2 using 20 mL disposable syringes with luer cone (Becton Dickinson Discardit® II) attached to 40 mm 18–20 G needles (Sterican®, Braun Melsungen AG). A total of 19.5 mL was collected, of which 0.5 mL was immediately transferred to a tube containing tripotassium ethylenediaminetetraacetic acid (K3-EDTA; BD Vacutainer, Becton Dickinson Discardit®) for hematological analyses. The remaining 20 mL was transferred to glass tubes with coagulation activators and PS granules to collect serum (Tapval®). Samples were refrigerated at 4 ${}^{\circ }$ C for transport and then centrifuged at 3500 rpm for 10 min (J.P. Selecta® centrifuge); the serum obtained was stored at -20 ${}^{\circ }$ C until analysis.

Hematological analyses of RBC count (RBC; 10 ${}^{6}$ / µ L), HB concentration (g dL ${}^{-1}$ ) and PCV (%) were performed using an automated ADVIA® 2120i analyzer (ADVIA® 2120i Siemens Healthcare Diagnostics Inc.).

Fe, Ferr ( µ g dL ${}^{-1}$ ), and T (mg dL ${}^{-1}$ ) concentrations were analyzed using a Spin 200E spectrophotometer using commercial house reagents based on colorimetry for Fe (FerroZine) and turbidimetry for Ferr (Latex) and T (Spinreact®, Barcelona, Spain). The sample detection limits for Fe and Ferr were 0.850 µ g dL ${}^{-1}$ to linearity limits of 1000 µ g dL ${}^{-1}$ and 5.04 µ g L ${}^{-1}$ , respectively. The intra- and interassay coefficients of variation (CVs) equated to 0.79 % and 3.17 % and to 5.1 % and 6.3 % for Fe and Ferr, respectively. TIBC (mg dL ${}^{-1}$ ) was measured by multiplying the values of T by the constant factor 1.27. UIBC (mg dL ${}^{-1}$ ) was calculated as the subtraction of TIBC and the serum Fe (Gottschalk et al., 2000). TSAT (%) was determined with the formula serum Fe/TIBC × 100 following the method of Persijin et al. (1971).

**Table 1:** Mean values ± SD of red blood cell, hemoglobin concentration, packed cell volume, and transferrin saturation during the 11 months of pregnancy in Spanish Purebred mares.

Months	RBC (10 ${}^{6}$ / µ L)	HB (g dL ${}^{-1}$ )	PCV (%)	TSAT (%)
1	8.64 ± 1.27	12.4 ± 1.23	42.4 ± 5.25	44.6 ± 6.24
2	8.68 ± 1.54	12.7 ± 1.58	40.7 ± 4.27	45.7 ± 6.89
3	8.97 ± 1.54	12.6 ± 1.32	43.0 ± 5.84	43.5 ± 6.99
4	8.44 ± 1.48	12.2 ± 1.18	43.1 ± 6.09	42.3 ± 5.89
5	8.30 ± 1.19	11.8 ± 0.68	41.5 ± 3.75	41.7 ± 7.43
6	9.07 ± 1.36	12.5 ± 1.68	45.2 ± 5.98	43.3 ± 6.76
7	9.42 ± 1.91	12.4 ± 1.33	44.6 ± 5.79	44.7 ± 5.00
8	9.65 ± 1.88	12.1 ± 0.92	44.2 ± 5.06	45.9 ± 5.97
9	8.39 ± 1.38	12.0 ± 0.98	41.8 ± 4.80	44.8 ± 8.29
10	8.09 ± 0,68	12.2 ± 1.01	40.9 ± 3.70	46.2 ± 9.20
11	8.76 ± 1.36	12.8 ± 1.10	42.5 ± 3.30	42.9 ± 7.65

RBC: red blood cell; HB: hemoglobin; PCV: packed cell volume; TSAT: transferrin saturation.

### 2.3 Statistical analysis

The effect of pregnancy on each parameter was analyzed using one-way ANOVA after data transformation to meet the conditions of normality and homoscedasticity using the Kolmogorov–Smirnov and Levene tests, respectively. The Tukey honestly significant difference (HSD) test was performed in those cases with statistically significant differences. Finally, the relationship between each parameter was determined by linear correlation analysis (Pearson's correlation). The level of significance was set at p<0.05 .

## 3 Results

RBC, HB, PCV, and TSAT did not change during the gestation. Table 1 includes the mean ± SD of RBC, HB, PCV, and TSAT values from the 1st to the 11th months of gestation. As recorded in Satué et al. (unpublished data), compared to the 1st month, Fe and Ferr concentrations increased from the 2nd month and the 3rd month ( p<0.05 ), remaining constantly higher from the 4th month to the 8th month ( p<0.05 ) and increasing progressively and significantly from the 9th month to the 11th month of gestation ( p<0.05 ).

**Figure 1:**
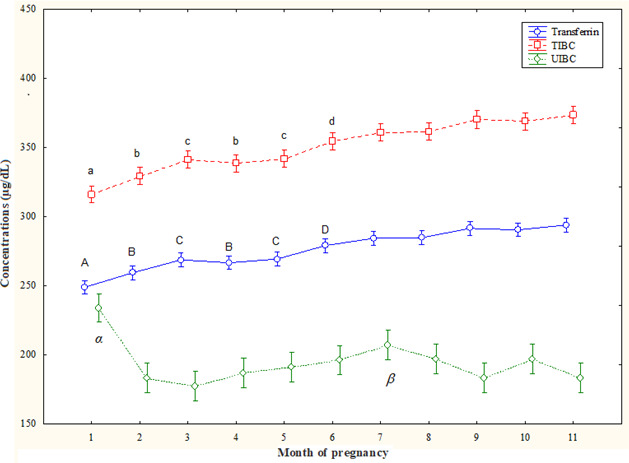
Circulating total iron-binding capacity (TIBC), unsaturated iron-binding capacity (UIBC), and transferrin (T) concentrations (mean ± SD) in pregnant Spanish Purebred mares. T – A: vs. 2–11 months; B: vs. 6–11 months; C: vs. 7–11 months; D: vs. 9 and 11 months ( p<0.05 ). TIBC – a: vs. 3–11 months; b: vs. 6–11 months; c: vs. 7–11 months; d: vs. 9 and 11 months ( p<0.05 ). UIBC – α : vs. 2–11 months; β : vs. 2 and 4 months ( p<0.05 ).

T concentrations increased from the 2nd month to the 11th month ( p<0.05 ) compared the 1st month and also from 6th month to the 11th month ( p<0.05 ) compared the 2nd month and the 4th month; T concentrations were lower at the 3rd month and the 5th month compared to values of the 7th–11th ( p<0.05 ) months. At the 6th month, they were lower ( p<0.05 ) than the values of the 9th month and the 11th month (Fig. 1).

TIBC concentrations increased along the 3rd–11th months ( p<0.05 ) compared to the 1st month and decreased at the 2nd month and the 4th month compared to 6th–11th months and also at the 3rd month and the 5th month compared to the 7th–11th months ( p<0.05 ). At the 6th month, they were lower ( p<0.05 ) than at the 9th month and the 11th month (Fig. 1).

UIBC concentrations decreased from the 2nd month to the 11th month ( p<0.05 ) compared to the 1st month, with lower values at the 7th month than at the 2nd month and the 4th month ( p<0.05 ) (Fig. 1).

**Table 2:** The correlation coefficients among all parameters considered in the study.

	HB	PVC	Fe	Ferr	T	TIBC	UIBC	TSAT
Parameters	(g dL ${}^{-1}$ )	(%)	( µ g dL ${}^{-1}$ )	( µ g dL ${}^{-1}$ )	(mg dL ${}^{-1}$ )	(mg dL ${}^{-1}$ )	(mg dL ${}^{-1}$ )	(%)
RBC (10 ${}^{6}$ / µ L)	0.68	0.80	0.07	0.02	0.03	0.03	0.00	0.09
HB (g dL ${}^{-1}$ )		0.69	0.13	0.11	0.14	-0.14	0.06	0.09
PVC (%)			0.13	0.04	0.00	0.00	0.04	0.15
Fe ( µ g dL ${}^{-1}$ )				0.21	0.40	0.40	0.04	0.94
Ferr ( µ g dL ${}^{-1}$ )					0.69	0.69	-0.69	-0.02
T (mg dL ${}^{-1}$ )						1.00	-0.09	0.07
TIBC (mg dL ${}^{-1}$ )							-0.09	0.07
UIBC (mg dL ${}^{-1}$ )								0.08

RBC: red blood cell; HB: hemoglobin; PCV: packed cell volume; Fe: iron; Ferr: ferritin; T: transferrin; TIBC: total iron-binding capacity; UIBC: unsaturated iron-binding capacity; TSAT: transferrin saturation.

## 4 Discussion

This study reports for the first time the relationships between erythrocyte parameters and the iron metabolic profile in the Spanish Purebred mare throughout gestation.

The most relevant results of the study have been the following: (1) the absence of modifications in the RBC, HB concentration, and PCV rules out the possibility of gestational anemia; (2) the increase in the concentrations of Fe, Ferr, T, and TIBC and the decrease in UIBC suggest that Fe requirements increase and are correctly met due to the demands imposed by the fetus and the maternal metabolism itself, ruling out Fe deficiency in the pregnant mare; and (3) the lack of modifications in TSAT could suggest the existence of a compensatory mechanism to avoid Fe overload in the pregnant mare.

The RBC count, HB concentration, and PCV did not change throughout gestation in Spanish Purebred mares. These results confirm those previously obtained in pregnant Carthusian mares (Satué et al., 2009), heavy draft mares (Aoki and Ishii, 2012), Quarter Horse mares, and Thoroughbred mares (Harvey et al., 1994). However, Faramarzi et al. (2018) showed significantly lower values of RBC, HB, and PCV in Andalusian, Quarter Horse, and Thoroughbred pregnant mares during the first and second trimesters compared to in non-pregnant mares. Likewise, in Marchador Mangalarga mares, Silva et al. (2019) reported the presence of hypochromia, macrocytosis, and anisocytosis associated with a decrease in RBC and PCV, concluding that pregnancy leads to macrocytic, hypochromic, low-intensity regenerative anemia. Interestingly, this evidence contrasts with that reported in bitches, in which non-regenerative anemia occurs according to the progression of pregnancy (Nivy et al., 2019). This decrease in RBC, HB, and PCV is widely documented in pregnant women (Li et al., 2017; Bakrim et al., 2018; Gebreweld et al., 2018; Philip et al., 2018). The putative mechanisms for these reductions in the RBC count include hemodilution, alterations in the erythrocyte half-life, and as possible vitamin–mineral deficiencies (Domosławska et al., 2013). It is known that placental and fetal development induces important changes in blood volume and red-cell mass. In fact, RBC mass increases by 15 %–20 % in response to increased production of erythropoietin. However, the increase in plasma volume is proportionally higher than the erythrocyte mass, being between 40 and 50 %. The hemodilution produced by the expansion of plasma volume in women is established in the second trimester of pregnancy to facilitate uteroplacental arterial flow and fetal growth (Kodali et al., 2008; Aguree and Gernand, 2019).

Plasma volume expansion is mediated by the direct action of progesterone and estrogen on the kidney, which causes renin release and consequent activation of angiotensin and aldosterone. Aldosterone in turn retains sodium in the kidneys and increases the volume of body water. Although there are no bibliographic reports regarding the expansion of blood volume in pregnant mares, the increase in activity of the renin angiotensin aldosterone system was previously documented (Satué and Domingo, 2011). This dilutional effect in women occurs more rapidly at the end of the second trimester and progressively reduces the RBC count (Fisher and Nemeth, 2017; Mutua et al., 2018). Therefore, to allow maximum availability of Fe for transfer across the placenta and maternal metabolism, intestinal absorption (Young et al., 2012; Sangkhae et al., 2020) and mobilization of reserve tissues in the liver and spleen, an increase in this period is expected (Ganz and Nemeth, 2012; Hubbard et al., 2013; Gao et al., 2015; Sangkhae et al., 2020). The decrease in erythrocyte half-life due to this emergency hematopoiesis in response to elevated erythropoietin levels is another one of the mechanisms involved in the decrease in RBC count and HB concentration during pregnancy in humans (Lurie and Mamet, 2000) and bitches (Nivy et al., 2019). Anemia during pregnancy is considered to be an adverse condition that can affect maternal–fetal health according to the WHO and is generally defined when HB values < 11 g dL ${}^{-1}$ (Wu et al., 2022).

Despite this evidence, in the Spanish Purebred mare, there is no tendency toward Fe deficiency or gestational anemia. In fact, free and stored Fe concentrations increased during the last 7 months, although without exceeding the reference intervals range of 105–277 µ g dL ${}^{-1}$ (Borges et al., 2007). This pattern shown by Fe and Ferr serum seems to indicate that the pregnant mare is quite efficient in regulating iron metabolism, particularly in advanced stages of gestation, to meet the demands imposed by the feto-placental unit and fetal development. Contrary to other studies (Faramarzi et al., 2018; Silva et al., 2019), the results provided in the RBC count in the concentration of HB, PVC, Fe, and Ferr levels could suggest that, in the Spanish Purebred mare, there is no the tendency toward anemia during pregnancy.

HB is the erythrocyte component that allows oxygen to reach the tissues and requires Fe for its synthesis. It appears that cytochromes and enzymes associated with tissue metabolism, as well as myoglobin, take priority in Fe supply, and as such, the first symptom of Fe deficiency is anemia. The anemia associated with iron deficiency is of the microcytic and hypochromic type. Currently, it is rare for diets fed to horses to cause iron deficiency anemia. In fact, Fe supplementation leads to Fe levels within the normal range in adult horses; although some show PCV at rest < 34 %, they do not indicate altered iron status. This scenario is common, and it is rare for a horse with a low PCV to respond to Fe supplementation with a concomitant increase in PCV. Therefore, clinical diet-based anemia in the horse is a rare entity (NRC, 2007). Most of the Fe that horses receive is derived from plants, supplements, or commercial products and from the soil, either from grazing or from soil contamination with hay. Thus, Fe deficiency is not a problem in adult horses if they have free access to soil apart from commercial forages and diets that contain more than sufficient amounts of this micronutrient. Fe absorption is estimated to be only about 15 % of the total amount due to saturation of absorption sites in the small intestine by the large amount of Fe available in normal equine diets (NRC, 2007).

Maternal and fetal Fe and Ferr levels are closely related (Upadhyaya et al., 2004; Adediran et al., 2013) such that placental adaptations mediated by increased vascularity and the transport and metabolism of amino acids, vitamins, and minerals are sufficient to attend to fetal growth during the last stage of gestation (Robles et al., 2018). Thus, a mare with adequate iron levels tends to produce fetuses and neonates with adequate iron levels as well (Upadhyaya et al., 2004).

The correlations between free and stored Fe (Ferr) were not close, suggesting that serum Fe levels are not entirely dependent on stores so that, perhaps, intestinal Fe absorption may be increased at the same time. The equine fetus indirectly receives Fe from the maternal circulation through a rapid and unidirectional process mediated by uteroferrin, an Fe transport protein necessary for the production of HB and other enzymatic cofactors at the fetal level (Wooding et al., 2000; Wooding and Fowden, 2006). Some studies have shown that uteroferrin is detectable from the time of endometrial cup development until day 309 of gestation, emphasizing the need for Fe transport across the placenta during this period (Ellenberger et al., 2008; Carter and Enders, 2013). The deposit of Fe in fetal tissues, which is minimal during the first 4 months, increases markedly after the fifth month so that Fe needs increase markedly in the final stages of gestation. In fact, 80 mg per kg body weight (BW) of Fe is necessary during the first 8 months of gestation in the mare, although this increases to 100 mg per kg BW in the last 2 months (NRC, 2007).

In women, rapid feto-placental growth, increased RBC mass, and hemodilution markedly reduced maternal Fe and Ferr concentrations between 12 and 25 weeks of gestation (Young et al., 2012; Milman et al., 2017; Vricella, 2017; Gao et al., 2019), which reflects the increased need for iron and promotes the release of stored Fe (Yang et al., 2019). Although the increase in Fe and Ferr physiologically characterizes pregnancy in the Spanish Purebred mare, in women the elevation of Ferr in the third trimester is associated with the risk of premature birth associated with lack of plasma volume expansion and intrauterine infection. Ferr is an acute phase protein that increases in response to infection and inflammation. In the presence of infection, macrophages produce inflammatory cytokines that generate reactive oxygen species, releasing free Fe from Ferr (Yokus et al., 2010).

Contrarily to what occurs in women and in the same way as in bitches (Nivy et al., 2019), TIBC concentrations increased progressively and significantly throughout gestation in the mare. Since TIBC is closely related to T concentration, increasing Fe requirements should also increase T concentration; however, TSAT did not change. This increase in T manages to maximize the available Fe in the organism. Given that TIBC and T increase, it is possible that, in order to maintain normal levels and without leading to an overload of circulating Fe, the TSAT does not change, which could indicate a regulation mechanism in the mare. The mean values of TSAT in pregnant mares are within the reference range reported for mammals in which the serum TSAT fluctuates between 20 % and 50 % (Anderson, 2002); so iron overload can be ruled out since an elevation of serum Fe and TSAT ( > 80 %) is indicative of iron overload (Theelen et al., 2019).

In contrast, TSAT increases in women in the first trimester and decreases throughout the second and third trimesters to levels lower than those in non-pregnant women (Yang et al., 2019). As the dynamics of TSAT are dependent on Fe and T concentrations, both parameters should increase in the first trimester, although Fe decreases and TIBC increases in the second and third trimesters (Yang et al., 2019). However, in women who develop Fe deficiency and anemia during pregnancy, TSAT decreases even though TIBC and UIBC increase significantly in the second and third trimesters compared to in the first trimester (Razza et al., 2011). Despite hemodilution, reduced Fe intake appears to be the cause of decreased HB, Fe, and TSAT during pregnancy in women (Milman et al., 2005).

In pregnant bitches, free Fe and TIBC concentrations are inversely correlated with acute phase reaction (APR) (McCown and Specht, 2011). Pregnancy in this species is characterized by a C-reactive protein-mediated APR, which peaks between 30 and 45 d after ovulation and declines abruptly at the end of pregnancy (Ulutas et al., 2009). Nivy et al. (2019) showed that serum Fe and TIBC levels decreased mid-pregnancy, coinciding with the APR peak, and increased at the end of gestation when the APR decreased. However, the increase in Fe and TIBC could not be justified in the mare based on APR. In fact, in a previous study carried out by these same researchers, it was not possible to confirm the existence of APR in the Spanish Purebred mare since the serum levels of type-A amyloid and C-reactive protein did not change throughout gestation (Satué et al., 2021).

Although the UIBC decreased in Spanish Purebred mares throughout gestation, the results regarding this parameter are contradictory in the female. While some report a decrease in UIBC, which could be a mechanism to ensure adequate fetal iron delivery based on the decrease in iron concentration with gestation (Amah-Tariah et al., 2011; Dangana et al., 2020), others report an increase as the age of gestation progressed (Razza et al., 2011). In addition, the negative correlation between UIBC and Fe in this study indicates that there is no iron depletion.

The increase in TIBC seems to suggest a greater generation of T for Fe transport in the mother. TIBC was measured by multiplying the T values by the constant factor 1.27, and UIBC was calculated as the subtraction of TIBC and serum iron; hence, the trend of these parameters was the same throughout the gestation period. This could indicate that bound and free Fe are sufficient and, therefore, that iron metabolism is efficient to meet the demands imposed by the fetus and the mother herself during pregnancy. It is unknown if the absorption or the type of diet supplied to the mare could increase in this period.

## 5 Conclusions

Contrary to what has been reported in other studies, the values of Fe, Ferr, RBC, HB, and PVC rule out Fe deficiency or gestational anemia in the mare. Given that TIBC indicates the Fe transport capacity based on the availability of T in the blood and that both parameters are closely related to the needs, it is possible to presume that pregnancy increases the baseline Fe requirements in the mare. The hematological parameters and iron status seem to indicate the pivotal efficiency in the transport, mobilization, and utilization of Fe during gestation in Spanish Purebred mares.

## Data Availability

The data are available from the corresponding author upon reasonable request.
